# Supplementary material for: Modeling glioblastoma heterogeneity as a dynamic network of cell states
Source: Mol Syst Biol. 2021 Sep 16;17(9):e10105. doi: 10.15252/msb.202010105 (PMC8444284; doi:10.15252/msb.202010105)
Supplement: Supplementary file 6 — Source Data for Figure 5 [file MSB-17-e10105-s004.zip › Figure5A_sourcedata/GSEA_3017/nosigdb_stateF.GseaPreranked.1622795015644/FELDMAN_2020_S-G2.html]

Details for gene set FELDMAN\_2020\_S-G2[GSEA]

|  || Dataset | state33017 |
| Phenotype | NoPhenotypeAvailable |
| Upregulated in class | na\_neg |
| GeneSet | FELDMAN\_2020\_S-G2 |
| Enrichment Score (ES) | -0.69124293 |
| Normalized Enrichment Score (NES) | -5.2650046 |
| Nominal p-value | 0.0 |
| FDR q-value | 0.0 |
| FWER p-Value | 0.0 |
Table: GSEA Results Summary

  

Fig 1: Enrichment plot: FELDMAN\_2020\_S-G2      
 Profile of the Running ES Score & Positions of GeneSet Members on the Rank Ordered List

  

| PROBE | GENE SYMBOL | GENE\_TITLE | RANK IN GENE LIST | RANK METRIC SCORE | RUNNING ES | CORE ENRICHMENT || 1 | CDKN1A |  |  | 4 | 2.089 | 0.0100 | No |
| 2 | CLU |  |  | 5 | 1.852 | 0.0207 | No |
| 3 | S100B |  |  | 20 | 1.167 | 0.0204 | No |
| 4 | SDCBP |  |  | 27 | 1.054 | 0.0235 | No |
| 5 | FTL |  |  | 32 | 1.036 | 0.0275 | No |
| 6 | RPS27L |  |  | 33 | 1.025 | 0.0334 | No |
| 7 | IFITM3 |  |  | 38 | 0.987 | 0.0371 | No |
| 8 | SAT1 |  |  | 45 | 0.957 | 0.0396 | No |
| 9 | SPARC |  |  | 52 | 0.929 | 0.0419 | No |
| 10 | SESN3 |  |  | 56 | 0.908 | 0.0457 | No |
| 11 | NDFIP1 |  |  | 74 | 0.814 | 0.0419 | No |
| 12 | MALAT1 |  |  | 90 | 0.773 | 0.0389 | No |
| 13 | ITM2B |  |  | 181 | 0.610 | -0.0023 | No |
| 14 | DDIT4 |  |  | 216 | 0.579 | -0.0159 | No |
| 15 | BTG1 |  |  | 222 | 0.574 | -0.0151 | No |
| 16 | TM7SF2 |  |  | 225 | 0.570 | -0.0128 | No |
| 17 | BEX4 |  |  | 242 | 0.559 | -0.0175 | No |
| 18 | MIAT |  |  | 263 | 0.542 | -0.0244 | No |
| 19 | ARL6IP5 |  |  | 264 | 0.542 | -0.0212 | No |
| 20 | SCD5 |  |  | 306 | 0.508 | -0.0387 | No |
| 21 | PTPRZ1 |  |  | 310 | 0.505 | -0.0373 | No |
| 22 | PLD3 |  |  | 337 | 0.494 | -0.0474 | No |
| 23 | EIF4A2 |  |  | 380 | 0.478 | -0.0655 | No |
| 24 | MAGED2 |  |  | 405 | 0.467 | -0.0747 | No |
| 25 | EEF1A1 |  |  | 417 | 0.464 | -0.0775 | No |
| 26 | PSAP |  |  | 418 | 0.463 | -0.0749 | No |
| 27 | PRCP |  |  | 426 | 0.460 | -0.0757 | No |
| 28 | TTYH1 |  |  | 457 | 0.448 | -0.0880 | No |
| 29 | BEX1 |  |  | 518 | 0.428 | -0.1154 | No |
| 30 | GPM6B |  |  | 553 | 0.413 | -0.1299 | No |
| 31 | IGFBP5 |  |  | 564 | 0.410 | -0.1325 | No |
| 32 | YPEL5 |  |  | 583 | 0.403 | -0.1392 | No |
| 33 | ATP6V1G1 |  |  | 609 | 0.395 | -0.1493 | No |
| 34 | FABP7 |  |  | 701 | 0.371 | -0.1924 | No |
| 35 | ID3 |  |  | 789 | 0.349 | -0.2337 | No |
| 36 | TMEM59 |  |  | 803 | 0.346 | -0.2381 | No |
| 37 | LAPTM4A |  |  | 854 | 0.335 | -0.2611 | No |
| 38 | HES4 |  |  | 950 | 0.317 | -0.3064 | No |
| 39 | SCRG1 |  |  | 1032 | 0.304 | -0.3450 | No |
| 40 | LYPD1 |  |  | 1094 | 0.293 | -0.3736 | No |
| 41 | ENC1 |  |  | 1287 | 0.270 | -0.4675 | No |
| 42 | RNF19A |  |  | 1429 | 0.255 | -0.5361 | No |
| 43 | POLD4 |  |  | 1430 | 0.255 | -0.5346 | No |
| 44 | RFC5 |  |  | 1473 | -0.250 | -0.5541 | No |
| 45 | SNRPG |  |  | 1486 | -0.254 | -0.5586 | No |
| 46 | RBBP8 |  |  | 1518 | -0.265 | -0.5724 | No |
| 47 | CEP78 |  |  | 1524 | -0.265 | -0.5734 | No |
| 48 | HSP90AA1 |  |  | 1535 | -0.268 | -0.5768 | No |
| 49 | RNPS1 |  |  | 1548 | -0.271 | -0.5812 | No |
| 50 | PSIP1 |  |  | 1564 | -0.275 | -0.5871 | No |
| 51 | PIN1 |  |  | 1565 | -0.275 | -0.5855 | No |
| 52 | PRKDC |  |  | 1594 | -0.285 | -0.5978 | No |
| 53 | HIST1H2AM |  |  | 1615 | -0.289 | -0.6061 | No |
| 54 | PARP2 |  |  | 1665 | -0.305 | -0.6287 | No |
| 55 | EIF4EBP1 |  |  | 1681 | -0.312 | -0.6343 | No |
| 56 | RBM17 |  |  | 1702 | -0.319 | -0.6425 | No |
| 57 | HAUS1 |  |  | 1708 | -0.322 | -0.6431 | No |
| 58 | PARP1 |  |  | 1713 | -0.323 | -0.6432 | No |
| 59 | PHLDA1 |  |  | 1718 | -0.324 | -0.6433 | No |
| 60 | UPP1 |  |  | 1726 | -0.328 | -0.6449 | No |
| 61 | MCM3 |  |  | 1738 | -0.335 | -0.6485 | No |
| 62 | HSPD1 |  |  | 1742 | -0.336 | -0.6480 | No |
| 63 | PAICS |  |  | 1757 | -0.342 | -0.6530 | No |
| 64 | RAB13 |  |  | 1761 | -0.345 | -0.6525 | No |
| 65 | RAN |  |  | 1772 | -0.350 | -0.6555 | No |
| 66 | PFDN2 |  |  | 1784 | -0.355 | -0.6589 | No |
| 67 | TMEM158 |  |  | 1803 | -0.362 | -0.6658 | No |
| 68 | WDR34 |  |  | 1806 | -0.365 | -0.6647 | No |
| 69 | C12orf75 |  |  | 1832 | -0.380 | -0.6749 | No |
| 70 | CDC45 |  |  | 1836 | -0.382 | -0.6742 | No |
| 71 | FBL |  |  | 1845 | -0.386 | -0.6760 | No |
| 72 | CDCA4 |  |  | 1852 | -0.391 | -0.6767 | No |
| 73 | EXOSC8 |  |  | 1865 | -0.397 | -0.6804 | No |
| 74 | SHCBP1 |  |  | 1886 | -0.412 | -0.6880 | No |
| 75 | RFC4 |  |  | 1887 | -0.413 | -0.6856 | No |
| 76 | SAE1 |  |  | 1893 | -0.417 | -0.6857 | No |
| 77 | ASF1B |  |  | 1897 | -0.419 | -0.6848 | No |
| 78 | MXD3 |  |  | 1905 | -0.428 | -0.6858 | No |
| 79 | HMGA1 |  |  | 1915 | -0.433 | -0.6878 | No |
| 80 | EIF5 |  |  | 1923 | -0.439 | -0.6887 | Yes |
| 81 | DIAPH3 |  |  | 1924 | -0.439 | -0.6862 | Yes |
| 82 | GGCT |  |  | 1930 | -0.442 | -0.6861 | Yes |
| 83 | GINS2 |  |  | 1932 | -0.445 | -0.6841 | Yes |
| 84 | MZT1 |  |  | 1936 | -0.447 | -0.6830 | Yes |
| 85 | SNRPB |  |  | 1937 | -0.447 | -0.6804 | Yes |
| 86 | HNRNPDL |  |  | 1940 | -0.448 | -0.6789 | Yes |
| 87 | RFC2 |  |  | 1954 | -0.457 | -0.6827 | Yes |
| 88 | EIF1AX |  |  | 1959 | -0.458 | -0.6820 | Yes |
| 89 | ZWILCH |  |  | 1962 | -0.461 | -0.6804 | Yes |
| 90 | DNMT1 |  |  | 1969 | -0.465 | -0.6807 | Yes |
| 91 | PTGES3 |  |  | 1970 | -0.467 | -0.6780 | Yes |
| 92 | PRELID1 |  |  | 1971 | -0.467 | -0.6753 | Yes |
| 93 | CMC2 |  |  | 1977 | -0.473 | -0.6751 | Yes |
| 94 | C19orf48 |  |  | 1979 | -0.474 | -0.6729 | Yes |
| 95 | POP7 |  |  | 1981 | -0.474 | -0.6706 | Yes |
| 96 | SUPT16H |  |  | 1986 | -0.479 | -0.6699 | Yes |
| 97 | NASP |  |  | 1988 | -0.481 | -0.6676 | Yes |
| 98 | PGP |  |  | 1990 | -0.482 | -0.6653 | Yes |
| 99 | TRA2B |  |  | 1998 | -0.490 | -0.6660 | Yes |
| 100 | PSMC3 |  |  | 1999 | -0.492 | -0.6631 | Yes |
| 101 | TK1 |  |  | 2003 | -0.497 | -0.6618 | Yes |
| 102 | LRR1 |  |  | 2004 | -0.497 | -0.6589 | Yes |
| 103 | TUBB6 |  |  | 2006 | -0.500 | -0.6565 | Yes |
| 104 | TOMM40 |  |  | 2008 | -0.501 | -0.6542 | Yes |
| 105 | SIVA1 |  |  | 2014 | -0.505 | -0.6537 | Yes |
| 106 | MAGOHB |  |  | 2015 | -0.505 | -0.6508 | Yes |
| 107 | HIRIP3 |  |  | 2017 | -0.508 | -0.6484 | Yes |
| 108 | FEN1 |  |  | 2018 | -0.511 | -0.6455 | Yes |
| 109 | SMC1A |  |  | 2021 | -0.513 | -0.6435 | Yes |
| 110 | RRM1 |  |  | 2023 | -0.516 | -0.6410 | Yes |
| 111 | RANBP1 |  |  | 2024 | -0.517 | -0.6381 | Yes |
| 112 | UBE2I |  |  | 2027 | -0.518 | -0.6361 | Yes |
| 113 | CACYBP |  |  | 2028 | -0.518 | -0.6331 | Yes |
| 114 | PDCD5 |  |  | 2031 | -0.520 | -0.6311 | Yes |
| 115 | IER2 |  |  | 2034 | -0.524 | -0.6291 | Yes |
| 116 | HELLS |  |  | 2038 | -0.528 | -0.6276 | Yes |
| 117 | SNRPA1 |  |  | 2040 | -0.528 | -0.6250 | Yes |
| 118 | CDCA3 |  |  | 2041 | -0.529 | -0.6220 | Yes |
| 119 | TRIP13 |  |  | 2044 | -0.530 | -0.6199 | Yes |
| 120 | NCL |  |  | 2045 | -0.532 | -0.6169 | Yes |
| 121 | KIF22 |  |  | 2053 | -0.541 | -0.6172 | Yes |
| 122 | CKLF |  |  | 2055 | -0.544 | -0.6146 | Yes |
| 123 | SRSF7 |  |  | 2056 | -0.544 | -0.6115 | Yes |
| 124 | CTNNAL1 |  |  | 2059 | -0.548 | -0.6093 | Yes |
| 125 | SNRPE |  |  | 2062 | -0.565 | -0.6071 | Yes |
| 126 | STMN1 |  |  | 2063 | -0.567 | -0.6038 | Yes |
| 127 | SPDL1 |  |  | 2064 | -0.569 | -0.6005 | Yes |
| 128 | PTMA |  |  | 2065 | -0.570 | -0.5973 | Yes |
| 129 | ZWINT |  |  | 2069 | -0.577 | -0.5954 | Yes |
| 130 | RTKN2 |  |  | 2071 | -0.579 | -0.5926 | Yes |
| 131 | CENPA |  |  | 2073 | -0.586 | -0.5897 | Yes |
| 132 | MND1 |  |  | 2074 | -0.586 | -0.5863 | Yes |
| 133 | LSM4 |  |  | 2076 | -0.587 | -0.5835 | Yes |
| 134 | SKA3 |  |  | 2077 | -0.590 | -0.5801 | Yes |
| 135 | BUB3 |  |  | 2078 | -0.596 | -0.5767 | Yes |
| 136 | AURKB |  |  | 2079 | -0.598 | -0.5732 | Yes |
| 137 | GGH |  |  | 2080 | -0.603 | -0.5697 | Yes |
| 138 | SRSF3 |  |  | 2081 | -0.606 | -0.5663 | Yes |
| 139 | CENPK |  |  | 2082 | -0.607 | -0.5628 | Yes |
| 140 | USP1 |  |  | 2084 | -0.612 | -0.5597 | Yes |
| 141 | SSRP1 |  |  | 2091 | -0.630 | -0.5591 | Yes |
| 142 | SET |  |  | 2095 | -0.639 | -0.5569 | Yes |
| 143 | MIS18A |  |  | 2096 | -0.639 | -0.5532 | Yes |
| 144 | DNAJC9 |  |  | 2097 | -0.641 | -0.5496 | Yes |
| 145 | HMGN1 |  |  | 2099 | -0.646 | -0.5463 | Yes |
| 146 | BRCA2 |  |  | 2100 | -0.650 | -0.5426 | Yes |
| 147 | SNRPD1 |  |  | 2105 | -0.653 | -0.5408 | Yes |
| 148 | PSMC3IP |  |  | 2106 | -0.659 | -0.5370 | Yes |
| 149 | KPNB1 |  |  | 2108 | -0.660 | -0.5337 | Yes |
| 150 | CKAP2L |  |  | 2109 | -0.663 | -0.5299 | Yes |
| 151 | RPA3 |  |  | 2112 | -0.678 | -0.5270 | Yes |
| 152 | CKS2 |  |  | 2115 | -0.694 | -0.5240 | Yes |
| 153 | TNFRSF12A |  |  | 2116 | -0.694 | -0.5200 | Yes |
| 154 | ANP32B |  |  | 2119 | -0.700 | -0.5170 | Yes |
| 155 | CDCA2 |  |  | 2120 | -0.705 | -0.5130 | Yes |
| 156 | SRSF2 |  |  | 2122 | -0.706 | -0.5094 | Yes |
| 157 | PA2G4 |  |  | 2123 | -0.707 | -0.5053 | Yes |
| 158 | CENPM |  |  | 2125 | -0.709 | -0.5018 | Yes |
| 159 | HNRNPD |  |  | 2126 | -0.713 | -0.4977 | Yes |
| 160 | RAD51AP1 |  |  | 2127 | -0.715 | -0.4935 | Yes |
| 161 | HNRNPAB |  |  | 2130 | -0.729 | -0.4903 | Yes |
| 162 | CKAP2 |  |  | 2131 | -0.729 | -0.4862 | Yes |
| 163 | ATAD5 |  |  | 2133 | -0.742 | -0.4824 | Yes |
| 164 | VRK1 |  |  | 2134 | -0.743 | -0.4781 | Yes |
| 165 | CMSS1 |  |  | 2140 | -0.772 | -0.4762 | Yes |
| 166 | NDC80 |  |  | 2142 | -0.775 | -0.4722 | Yes |
| 167 | H2AFV |  |  | 2143 | -0.779 | -0.4677 | Yes |
| 168 | FBXO5 |  |  | 2144 | -0.779 | -0.4632 | Yes |
| 169 | KIF23 |  |  | 2145 | -0.782 | -0.4587 | Yes |
| 170 | GMNN |  |  | 2146 | -0.785 | -0.4542 | Yes |
| 171 | MELK |  |  | 2148 | -0.786 | -0.4502 | Yes |
| 172 | ANLN |  |  | 2149 | -0.797 | -0.4456 | Yes |
| 173 | SMC3 |  |  | 2150 | -0.800 | -0.4410 | Yes |
| 174 | KIFC1 |  |  | 2151 | -0.802 | -0.4364 | Yes |
| 175 | DEPDC1 |  |  | 2152 | -0.809 | -0.4318 | Yes |
| 176 | DEK |  |  | 2154 | -0.817 | -0.4276 | Yes |
| 177 | DUT |  |  | 2155 | -0.824 | -0.4228 | Yes |
| 178 | TUBB |  |  | 2157 | -0.831 | -0.4185 | Yes |
| 179 | MCM7 |  |  | 2160 | -0.839 | -0.4147 | Yes |
| 180 | TUBB4B |  |  | 2162 | -0.843 | -0.4104 | Yes |
| 181 | KIF20B |  |  | 2165 | -0.852 | -0.4064 | Yes |
| 182 | ARHGAP11A |  |  | 2167 | -0.855 | -0.4020 | Yes |
| 183 | DTYMK |  |  | 2168 | -0.858 | -0.3971 | Yes |
| 184 | NUF2 |  |  | 2176 | -0.877 | -0.3955 | Yes |
| 185 | CEP55 |  |  | 2177 | -0.883 | -0.3905 | Yes |
| 186 | KPNA2 |  |  | 2178 | -0.894 | -0.3853 | Yes |
| 187 | CCDC34 |  |  | 2180 | -0.898 | -0.3806 | Yes |
| 188 | CCNA2 |  |  | 2181 | -0.902 | -0.3755 | Yes |
| 189 | MIS18BP1 |  |  | 2183 | -0.903 | -0.3708 | Yes |
| 190 | SMS |  |  | 2184 | -0.910 | -0.3655 | Yes |
| 191 | KIF4A |  |  | 2186 | -0.921 | -0.3607 | Yes |
| 192 | ORC6 |  |  | 2188 | -0.937 | -0.3558 | Yes |
| 193 | CENPH |  |  | 2191 | -0.943 | -0.3514 | Yes |
| 194 | CENPW |  |  | 2192 | -0.943 | -0.3460 | Yes |
| 195 | TUBA1B |  |  | 2193 | -0.947 | -0.3406 | Yes |
| 196 | LMNB1 |  |  | 2194 | -0.950 | -0.3351 | Yes |
| 197 | HJURP |  |  | 2195 | -0.962 | -0.3296 | Yes |
| 198 | UBE2T |  |  | 2196 | -0.965 | -0.3240 | Yes |
| 199 | HIST1H2AH |  |  | 2197 | -0.965 | -0.3185 | Yes |
| 200 | CKS1B |  |  | 2201 | -0.987 | -0.3143 | Yes |
| 201 | HIST1H1E |  |  | 2202 | -0.992 | -0.3086 | Yes |
| 202 | ESCO2 |  |  | 2204 | -1.004 | -0.3033 | Yes |
| 203 | SMC2 |  |  | 2205 | -1.006 | -0.2975 | Yes |
| 204 | SPC25 |  |  | 2208 | -1.019 | -0.2927 | Yes |
| 205 | CENPE |  |  | 2211 | -1.036 | -0.2877 | Yes |
| 206 | DHFR |  |  | 2212 | -1.040 | -0.2817 | Yes |
| 207 | NUDT1 |  |  | 2214 | -1.054 | -0.2761 | Yes |
| 208 | MYBL2 |  |  | 2215 | -1.058 | -0.2701 | Yes |
| 209 | CLSPN |  |  | 2216 | -1.060 | -0.2640 | Yes |
| 210 | CENPN |  |  | 2217 | -1.079 | -0.2578 | Yes |
| 211 | UBE2C |  |  | 2218 | -1.081 | -0.2515 | Yes |
| 212 | CENPU |  |  | 2219 | -1.088 | -0.2453 | Yes |
| 213 | H2AFZ |  |  | 2220 | -1.090 | -0.2390 | Yes |
| 214 | HMGB1 |  |  | 2221 | -1.116 | -0.2326 | Yes |
| 215 | NCAPG |  |  | 2222 | -1.122 | -0.2261 | Yes |
| 216 | H2AFX |  |  | 2223 | -1.146 | -0.2196 | Yes |
| 217 | CDK1 |  |  | 2225 | -1.153 | -0.2134 | Yes |
| 218 | PTTG1 |  |  | 2226 | -1.198 | -0.2065 | Yes |
| 219 | MAD2L1 |  |  | 2228 | -1.204 | -0.2001 | Yes |
| 220 | UBE2S |  |  | 2229 | -1.205 | -0.1932 | Yes |
| 221 | GTSE1 |  |  | 2230 | -1.220 | -0.1862 | Yes |
| 222 | HIST2H2AC |  |  | 2231 | -1.231 | -0.1791 | Yes |
| 223 | DBF4 |  |  | 2232 | -1.247 | -0.1719 | Yes |
| 224 | HMGN2 |  |  | 2233 | -1.267 | -0.1646 | Yes |
| 225 | RRM2 |  |  | 2234 | -1.268 | -0.1573 | Yes |
| 226 | SMC4 |  |  | 2236 | -1.281 | -0.1505 | Yes |
| 227 | PBK |  |  | 2237 | -1.325 | -0.1428 | Yes |
| 228 | ATAD2 |  |  | 2238 | -1.381 | -0.1349 | Yes |
| 229 | TMPO |  |  | 2239 | -1.382 | -0.1270 | Yes |
| 230 | PRC1 |  |  | 2240 | -1.397 | -0.1189 | Yes |
| 231 | HMGB2 |  |  | 2241 | -1.469 | -0.1105 | Yes |
| 232 | BIRC5 |  |  | 2242 | -1.469 | -0.1020 | Yes |
| 233 | HIST1H1C |  |  | 2243 | -1.485 | -0.0935 | Yes |
| 234 | ASPM |  |  | 2244 | -1.490 | -0.0849 | Yes |
| 235 | TOP2A |  |  | 2246 | -1.562 | -0.0764 | Yes |
| 236 | NUSAP1 |  |  | 2247 | -1.600 | -0.0672 | Yes |
| 237 | TPX2 |  |  | 2248 | -1.609 | -0.0580 | Yes |
| 238 | CENPF |  |  | 2249 | -1.691 | -0.0483 | Yes |
| 239 | HIST1H1D |  |  | 2250 | -1.720 | -0.0384 | Yes |
| 240 | HIST1H1B |  |  | 2251 | -1.796 | -0.0280 | Yes |
| 241 | MKI67 |  |  | 2252 | -1.892 | -0.0172 | Yes |
| 242 | HIST1H4C |  |  | 2253 | -2.984 | -0.0000 | Yes |
Table: GSEA details [plain text format]

  

Fig 2: FELDMAN\_2020\_S-G2: Random ES distribution      
 Gene set null distribution of ES for **FELDMAN\_2020\_S-G2**
